# Examining the Potential of Blockchain Technology to Meet the Needs of 21st-Century Japanese Health Care: Viewpoint on Use Cases and Policy

**DOI:** 10.2196/13649

**Published:** 2020-01-09

**Authors:** Tim Mackey, Hirofumi Bekki, Tokio Matsuzaki, Hiroshi Mizushima

**Affiliations:** 1 Department of Anesthesiology and Division of Global Public Health University of California San Diego School of Medicine La Jolla, CA United States; 2 Global Health Policy Institute La Jolla, CA United States; 3 BlockLAB San Diego Super Computer Center La Jolla, CA United States; 4 Department of Orthopedic Surgery Kyushu University Fukuoka Japan; 5 Japan Biodesign Program Department of Cardiac Surgery The University of Tokyo Tokyo Japan; 6 Center for Public Health Informatics National Institute of Public Health Saitama Japan; 7 Japanese Medical Blockchain Association Tokyo Japan

**Keywords:** information storage and retrieval, blockchain, Japan, aging, health informatics, health policy, global health

## Abstract

Japan is undergoing a major population health transition as its society ages, and it continues to experience low birth rates. An aging Japan will bring new challenges to its public health system, highlighted as a model for universal health coverage (UHC) around the world. Specific challenges Japan’s health care system will face include an increase in national public health expenditures, higher demand for health care services, acute need for elder and long-term care, shortage of health care workers, and disparities between health care access in rural versus urban areas. Blockchain technology has the potential to address some of these challenges, but only if a health blockchain is conceptualized, designed, localized, and deployed in a way that is compatible with Japan’s centralized UHC-centric public health system. Blockchain solutions must also be adaptive to opportunities and barriers unique to Japan’s national health and innovation policy, including its regulatory sandbox system, while also seeking to learn from blockchain adoption in the private sector and in other countries. This viewpoint outlines the major opportunities and potential challenges to blockchain adoption for the future of Japan’s health care.

## Introduction

### Background

Blockchain technology is increasingly gaining interest around the world and is poised to be a technology that rapidly globalizes, now over a decade since the author under the Japanese name Satoshi Nakamoto first published the bitcoin white paper that led to popularization of the technology. Fundamentally, blockchain is a form of distributed digital ledger technology used to share and store data in a decentralized manner [1]. *Blocks* of data are secured through cryptography so that a *chain* of blocks is created that provides the provenance of any given transaction and also makes the records tamper evident [2]. In the context of health care, a *health* blockchain can enable better trust, security, management, and transparency of health care data, processes, and transactions and is actively being explored as a potential tool to improve the delivery of health care in several countries [3].

Despite growing attention, investment, and ongoing efforts toward commercialization, blockchain technology is still in its early adoption phases, particularly when comparing the health care industry to other sectors such as cryptocurrency markets, financial technology, and blockchain for logistics [4]. Importantly, blockchain solutions need to be tailored to the challenges unique to health care, including issues related to patient and data privacy, coordinating care across multiple actors (including hospital, payers, health care providers, pharmaceutical and device manufacturers, health technology providers, and patients), ensuring appropriate sharing, and maintaining integrity of health care data, while also being responsive to regulatory complexity [3,5].

With its potential to transform different industry verticals, several proofs of concept, pilot projects, and solutions in transition between development and production are being explored by the private sector (including large to midsize companies and startups), consortiums, and national governments who are specifically focused on health care [3,6,7]. Beyond tailoring blockchain solution design to the health care sector, blockchain use cases also need to be localized to the specific community and population health needs faced by an individual country and also be compatible with its national health system design.

### Localizing Blockchain for Health and Japan

The need to localize blockchain solutions includes countries such as Japan, the third largest economy in the world (by nominal gross domestic product [GDP]), which has recently seen increased activity around assessing blockchain applications in the health care sector that are specific to its unique and emerging clinical and public health challenges [8]. Specifically, Japan operates a robust public health system, characterized by universal health coverage (UHC), which has long been touted by the Japanese government as first class in terms of access, quality, and low cost [9].

Despite Japan’s strong public health system and commitment to UHC, its 21st-century health care challenges are historically unique. Central to these challenges is a demographic shift that is underway called *koreikashakai* (translated as *population aging society*), which has resulted in Japan becoming the world’s oldest country (with 27.7% [n=35.15 million] of its population aged older than 65 years) [10]. The resulting *upside down* demographic pyramid (precipitated by low birth rate and high life expectancy) will bring new economic challenges (eg, stabilizing funding of national pensions with concomitant need for growing availability of public services) coupled with increased strain on national health care capacity. Specific challenges include increased national public health expenditures, higher demand for health care services lacking appropriate cost controls, acute need for elder and long-term care, lack of availability and shortage of health care workers (including nurses and caregivers), and continuing disparities between health care access in rural versus urban areas [11-13].

Some of these challenges are becoming more acute, with recent data indicating that medical-related spending is growing in Japan [14]. Furthermore, although Japan operates a national health insurance system and a centralized social security and tax number system (known as *My Number system*), adoption of centralized health informatics infrastructure is lagging. This includes electronic medical records, where, in 2017, the adoption rate in Japan was reported as only 34.4% according to the Japan Country Commercial Guide published by export.gov, although implementation rates are projected to increase [15]. Hence, the digitization of Japan’s health system, which could enable creation of centralized health care data management and decision making remains limited, despite an acute need to lower health care–related administrative costs and provide more efficient access for increasing demand of health care services.

All these factors give rise to promising prospects for blockchain solutions to address specific needs of Japan’s health care system, but only if they are conceptualized, designed, and deployed to align with the country’s national health identity that focuses on a UHC-centric public health system. In response, this viewpoint outlines the unique opportunities and challenges faced by Japan’s current and future health care system, examines how these challenges can be addressed by blockchain technology, and also discusses emerging Japanese public policy on technology and whether it can encourage blockchain adoption.

## Japan’s Current and Future Health Care Challenges

First, it is important to understand the characteristics of Japan’s health care system and the current challenges it faces today and in the future. Japan’s health care system is based on a centralized UHC public insurance system with care provisioned by a network of more than 4000 public and private payers [16]. Residents of Japan are required to have health insurance coverage and receive coverage through Employees’ Health Insurance (*Kenko-Hoken*) or the national health insurance system (*Kokumin-Kenko-Hoken*). Citizens without insurance coverage from employers can participate in the national health insurance program. Depending on the total income and age of the insured, the ratio of medical fees patients pay differs from 0% to 30%, with the government paying the remaining fees [17]. The national health insurance system is based on fiscal resources generated by a combination of employee and employer contributions, cost sharing by patients, and subsidization by the government, all factors that are impacted by demographic changes.

The impact of demographic changes on public health care access, provisioning, and financing is central to technology development. Japan is now a rapidly aging society with the number of elderly people (aged older than 65 years) quadrupling in the last 40 years with some projections also suggesting that this will lead to a long period of overall population decline [18]. In fact, the number of elderly is expected to peak at a staggering 39.35 million by 2042, which will represent more than one in three people being elderly [10,19]. Conversely, the population of the labor force (aged 15-64 years) has been decreasing since 1995 when 87 million people were included in this group but, in 2016, only comprised 76 million people [10]. The labor force is further estimated to shrink to 68 million in 2030, also coinciding with an increase in nonworking pensioners [10]. This will lead to a decrease in working age individuals and employers who can contribute payroll taxes to the health system to fund public health programs [10]. It will also shift the risk pool of enrollees to more expensive patients who require higher frequency visits, long-term care, and more complex health interventions.

All these demographic trends point to a trifecta of health system shocks, including increased utilization, rising national medical costs, and decreases in health care financing relative to population and pensioner changes [10]. In 2015, Japan’s total health spending accounted for 11.2% of its GDP equating to 42.3 trillion yen and is now ranked third in total health spending out of 35 Organisation for Economic Co-operation and Development (OECD) countries [14,20]. Japan’s increase in health spending of GDP also reflects an overall trend of steadily increasing global expenditures on health care, including a projected 9% of GDP allocated globally to health spending by 2040 [21]. Japan’s elderly care health expenditure is also projected to rapidly increase, estimated to peak in the next few decades and then continue increasing until 2065 [22]. Given these characteristics of the *aging of Japan* and its public health system design, it is expected that increased costs related to a growing burden of chronic diseases and associated high-cost medical interventions and technologies will result in a health care funding gap of approximately 44 trillion yen by 2035 [16].

These factors are also exacerbated by suboptimal health care utilization in the country. For example, Japanese patients tend to go to outpatient clinics more often than in other OECD countries; Japanese physicians see approximately twice as many patients annually compared with other countries, and the length of hospital stay is very high [14]. The provisioning of unnecessary health care services, which includes higher volume or higher costs, is also something Japan is struggling to tackle. In addition, Japan aggressively introduces and uses advanced medical devices and new health technology, which increase the cost of diagnosis and disease management [23].

Expensive medical services include radiographic examination procedures, such as computed tomography and magnetic resonance imaging, which result in Japanese patients having higher exposure to radiation compared with other developed countries and concomitantly higher costs because of the frequency of these procedures [24]. Overutilization is incentivized by a fee-for-service model coupled with a national pricing structure that is meant to control costs but not utlization [25]. Overutilization may also be related to lack of comprehensive facility accreditation, as, currently, the Japan Council for Quality Health Care (JCQHC; established in 1995) acts as third-party accreditor to evaluate the functions of medical institutions, but only 26.2% (n=2192) of hospitals nationwide are certified and reviewed by the JCQHC [26].

In addition, with the increasing number of elderly patients, more physicians and other health care professionals (including nurses and caregivers) will be needed in the health care workforce [11,12]. According to a survey by the National Institute of Population and Social Security Research in Japan, the rate of total number of medical doctors was 2.4 per 1000 people in 2010, which is fewer than the average of other OECD countries [11]. Physician shortages are also impacted by a system where physicians can decide their specialties freely regardless of grades or achievements. In terms of board certification, historically, Japan has not set a limitation on the number of doctors in each department that approves these specialties leading to specialty imbalances. In addition to physicians, the Ministry of Health, Labour and Welfare (MHLW) has also reported a gap of 2 million nursing personnel as would be required by 2025 to meet health care utilization demands [12]. Furthermore, Japan’s immigration policies and bilateral trade agreements have also negatively impacted availability to foreign nursing and caregiver personnel [27,28].

Finally, there exists a growing gap of medical care coverage between rural and urban communities. In 2017, the reporting agency Nikkei, Inc, conducted an analysis on government data and reported that the mortality rate from acute myocardial infarction differed 4 to 5 times depending on whether a patient resided in a rural or urban community [13]. These data suggest that the quality of medical care may experience variation depending on geographic location (including rural vs urban) and that these differences can manifest in different communities even in the same prefecture.

In summary, Japan’s current and future health care challenges are largely driven by its rapidly aging demographic and how it impacts national health care expenditure, utilization, and workforce demands in a system that champions UHC. Importantly, other countries are also experiencing some of these challenges including aging populations and lower birth rates, higher burden of chronic diseases (including lower income countries experiencing an epidemiological shift from communicable to noncommunicable diseases), and lack of access to rural health care, but perhaps not to the same extent Japan is facing because of its rapidly changing yet still largely homogenous demographics (in 2018, Japan hit a record of 2.497 million foreigners, but this still represents only 1.99% of the entire population) [29].

Although technology is not the panacea for all these problems, blockchain in conjunction with other digital health solutions has the potential to lower health care costs, enable extension and broader access to health care services, combat health care fraud, and increase the efficiency of the health care workforce. We explore some existing and potential health blockchain use cases for Japan below.

## Availability of Data and Materials

Data associated with our multilingual literature and legal, and policy review are available via information in references.

## Blockchain: A Potential Solution for Japan’s Future Health Care Landscape?

**Figure 1:**
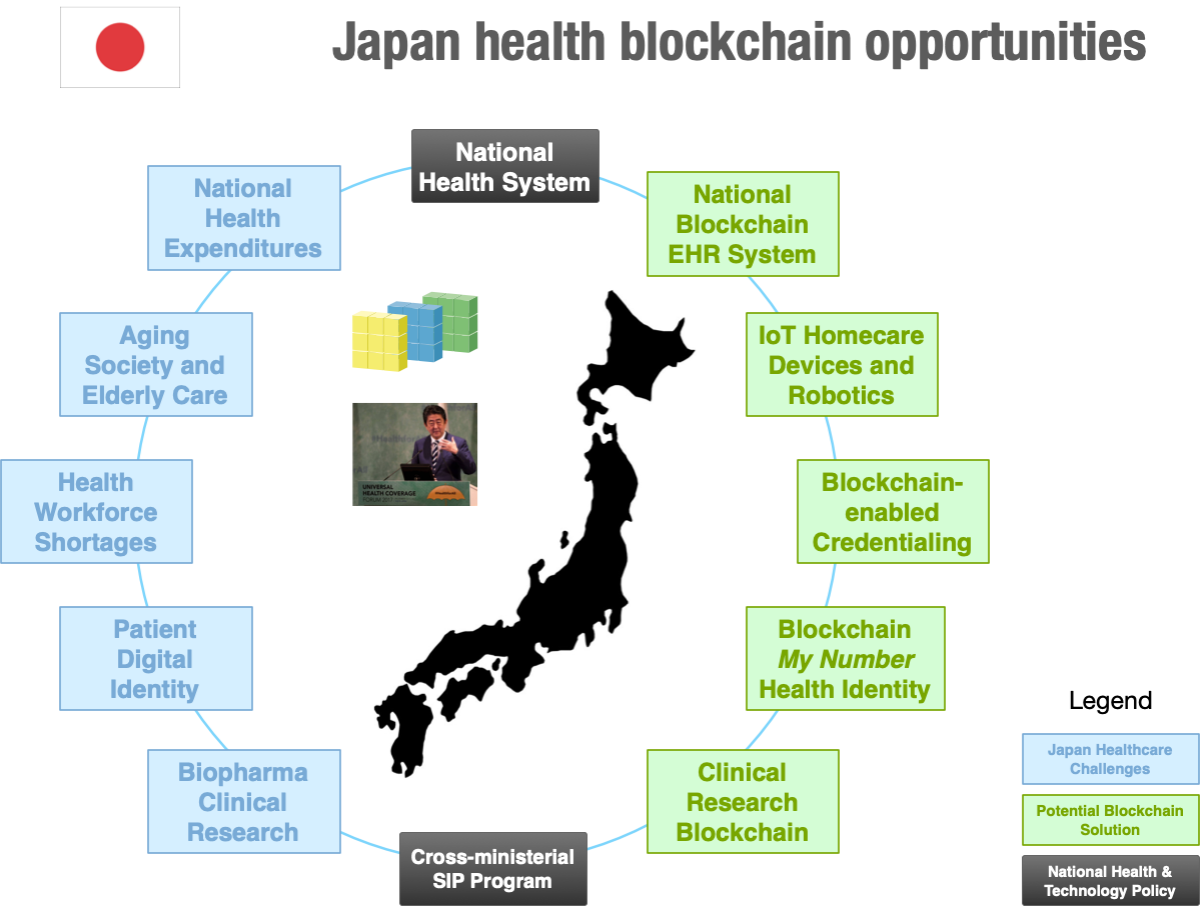
Summary of blockchain potential for the Japanese health care system. EHR: electronic health record; IoT: Internet of Things; SIP: Cross-ministerial Strategic Innovation Promotion Program.

Given the unique current and future challenges faced by Japan’s national health system, what challenge areas are blockchain technology solutions uniquely positioned to offer real-world solutions? The answer to that question resides in mapping use cases to Japan’s UHC-based health care system, examining how they could be localized, and also taking into account the national policy and health care information technology (IT) architecture of a centralized public health system (see Figure 1 for a summary of thematic blockchain opportunities).

Current and future adoption of blockchain solutions in Japan may also originate from use cases that do not directly address national health system priorities. Instead, these use cases might target Japan’s private sector and commercial needs, particularly relevant as Japan is the world’s third largest pharmaceutical market and one of the world’s largest markets for medical devices [30,31]. We explore select Japanese private and public sector blockchain health care use cases in the following sections, with some examples of use cases which are summarized in Multimedia Appendix 1.

### Private Sector Blockchain Use Cases

Translating blockchain solutions to the Japanese system context can first start by examining many of the leading use cases for health and life science blockchain deployments in other markets. These include management of electronic health records (EHRs), optimizing the performance of clinical trials and enhancing the integrity of the health and pharmaceutical supply chain [3,32-36]. Many of these use cases are more general to improving the competitiveness of Japan’s biotechnology, pharmaceutical, medical device, and life sciences private sector industries and not specific to its national health care system but still merit examination and could drive public sector adoption.

A leading health care blockchain area of development is clinical research blockchains that can produce verifiable data associated with patient recruitment, enhance sharing of clinical research data with patients and clinical trial study protocol data management, and, when provided a set of core defined metadata, can help ensure clinical trial integrity, transparency, and auditability to regulators [37,38]. Clinical trial blockchain solutions and consortiums are increasingly becoming more active, with major pharmaceutical manufacturers such as Boehringer Ingelheim, Pfizer, Amgen, Sanofi, and Bayer announcing initiatives in this area [3,39].

The Japanese startup company, Susmed Inc, recently announced a pilot project that has been certified by MHLW and the Ministry of Economy, Trade, and Industry for securing reliable clinical data monitoring using blockchain technology and mobile health (mHealth; mobile phone) apps [40,41]. In addition, the Japanese pharmaceutical manufacturer, Takeda, announced it was co-chairing with Microsoft and ERORDIS-Rare Disease Europe the *Global Commission to End the Diagnostic Odyssey for Children* aimed at addressing barriers to developing diagnostics for patients with rare diseases, which includes a pilot project examining the use of blockchain to develop secure patient registries and a rare disease patient passport [42]. A clinical research blockchain system could have also helped to avoid the 2014 arrest of a former Japanese employee of Novartis who was accused of falsifying clinical data for the popular hypertension drug, valsartan [43].

Similarly, blockchain solutions that are being developed to better secure the integrity of pharmaceutical supply chain in the United States and the European Union may also have utility in addressing infiltration of substandard and falsified medicines in Japan’s health system and also assist Japanese pharmaceutical manufacturers in securing international distribution of their own products [3]. Several companies are currently developing blockchain solutions for the pharmaceutical supply chain, including MediLedger, IBM, and SAP. They are primarily examining how blockchain can be used to establish provenance of drug pedigree and how it can enable regulatory adherence to national track-and-trace requirements, and they have also examined the potential utility of combating infiltration of substandard and falsified drugs [3,44].

In fact, there have been reports of counterfeit versions of erectile dysfunction drugs and weight loss pills being imported via internet sales and even counterfeit hepatitis C medication being detected in legitimate Japanese pharmacy chains [45-47]. Furthermore, the MHLW has established a *Suspicious Drugs Reporting Network* demonstrating growing public concern over the issue, particularly in the context of illegal internet pharmacies that operate outside the legitimate supply chain [48]. In addition, unrelated to the pharmaceutical supply chain but relevant to supply chain integrity, IBM and Asahi Refining (part of Japanese company Asahi Holdings) are part of a consortium called the TrustChain Initiative that will develop a blockchain network for establishing provenance for jewelry tracing that could show utility for health care commodities [49].

### Blockchain Solutions for Japan’s National Health System Challenges

Beyond commercially viable blockchain solutions for Japan’s private sector, some blockchain use cases map directly to Japan’s future demographically led health care challenges as previously summarized*.* These use cases include (1) blockchain-based EHR systems to improve health care billing, utilization, and reducing waste to ensure efficiency in national health care administration and financing; (2) blockchain-enabled provider and patient directories while also unifying services together under a blockchain digital identity tied to national social security system records; and (3) blockchain integration with Internet of Medical Things (IoMT) to address the aging society by enabling secure and verifiable home care delivery and telehealth.

### Blockchain-Based Medical Record Systems

First, although Japan is a public health system, electronic records are generally managed by individual institutions and are housed locally on disparate systems operated by private firms, not in a centralized database by the national government. Hence, patients’ data and health identity are scattered across different providers, which impedes access and portability, while also limiting big data analysis for insights into population health trends. The Japanese government also currently prohibits mixed billing, which consists of private and public health insurance for 1 condition; therefore, patients often have no choice but to select the treatment covered by their insurance [50]. This policy decision means future EHR and reimbursement blockchains will need to be responsive to centralized financing, although health care systems and providers themselves may be decentralized in operating and managing their health IT systems and data. For example, even radiographic examinations performed in Japan on the same day can be conducted at different provider locations, with the images residing in different databases. Differences in health system design and policy means that there will be multiple trade-offs in the design, development, and implementation of blockchain EHR systems related to utility of the system, security, and scalability that could be informed by the Model National Health Service blockchain as proposed by O’Donoghue et al [51].

Importantly, lagging adoption of health information exchange (HIE) despite a system that is centralized and is single payer provides an opportunity for blockchain to act as an intermediary or locator service to make queries of traditional database-bound medical records from different providers. Although there are signs that HIE between different health care systems in Japan is spreading, a privacy framework and mandated EHR data sharing across all 47 prefectures remains absent [52-54]. This problem seems well situated for blockchain adoption, including exploring managing patient digital data and identity through a shared distributed ledger that can be accessed by providers and is tied to the *My Number* social security and national tax number ID system. Despite the potential promise of blockchain-enabled HIE, concerns about patient privacy, coordination, opt-in to applications, and specific information exchange scenarios need to be further studied [55].

Examples that Japan could examine to assess the initial viability of a national health blockchain EHR system include a recent announcement by Taipei Medical University of the launch of a blockchain solution to improve patient referral services and integrate health care networks to enable better access to medical records [56]. The system includes participation from more than 100 community-based clinics, uses smart contracts, and enables access to EHR data [56,57]. Importantly, Taiwan also operates a national single-payer publicly funded health system under its National Health Insurance that covers 99% of the population and involves both public and private providers [58]. In this sense, it operates a health care system with many similarities to Japan, including challenges related to health data management and sharing, and hence could be informative to Japan’s own blockchain development around EHRs [58].

Other countries, such as Estonia that has adopted widescale use of electronic health (eHealth) approaches to its national health care system (including a Web-based eHealth record, electronic ID card, and electronic patient portal), are also assessing blockchain for purposes of maintaining security and integrity of health records and could also be informative [59,60]. There are also several companies, initiatives, consortiums, and research groups looking at blockchain EHR integration in the United States. This includes MedRec, an open-source platform that uses blockchain and smart contracts to create a record of patient-provider interactions and access and viewing permissions of medical records [61,62].

### Blockchain Directories and Unification Under My Number System

Another concrete example of blockchain use in health care data is blockchain-based provider data management and directory systems that attempt to reconcile health care provider credentialing and national medical licenses, which can be subject to fraud and error, including cases of identity fraud involving those pretending to be physicians in Japan [3,63,64]. Hence, a blockchain provider directory can provide a single source of verified data that can be better shared across different hospitals and payers, such as the one currently being explored by the Synaptic Health Alliance (which includes large US health care organizations such as Aetna, UnitedHealth, and Humana) [65,66]. Such a system could also be integrated with existing efforts by the Japan Medical Association to digitize medical credentialing under its JMA Electronic Certification Center using smart cards, electronic signatures, and identity authentication [64].

Compared with the complexity of the US health care system, which includes both public and private payers and providers, instituting a provider blockchain directory in Japan’s single-payer UHC structure should be a more straightforward task. A blockchain-based provider directory also opens the door for the development of a public national patient-centered directory with health care access verified by the *My Number* system. This could provide patients and providers with EHR verification (tied to validated *My Number* digital identity), recording and auditability of requests for data access, portability of health care data, enhance verification of health care claims, and potentially improve the continuity of care as pointers to EHRs could be linked to the *My Number* system across different Japanese health care providers [67].

Importantly, this design could adapt well to Japan’s current decentralized network of health care IT management where EHRs may continue to reside within each individual health care organization with hashed pointers enabling sharing in a secure and distributed fashion while also encouraging patient-driven interoperability [62,68]. Access could be based on principles of patient-centered permissions under a *My Number* digital identity and/or corresponding digital wallet, effectively mitigating the possibility for social welfare or health care fraud (eg, recently a woman in her 70s was defrauded several million yen in a scam associated with her My Number system ID) [69]. Finally, health data could also be aggregated and deidentified for population health research purposes [67].

### Integrating Blockchain to Japan’s Internet of Things Ecosystem

Integrating blockchain into the Internet of Things (IoT) and more specifically IoMT to better enable home care and telehealth services will also be a leading future use case in Japan, especially given the rise of connected medical devices and the ubiquitous use of other tools that can enable medical applications (such as mobile phones). Specifically, Japan is a country with widespread mobile phone adoption with 92% of adults reporting they own a mobile device according to a survey by the Pew Research Center among 30,133 people in 27 countries in 2018 [70]. Hence, increased health care utilization in the inpatient setting because of growth in the number of elderly patients and continued shortages of health care workers presents opportunities for expansion of Japan’s collective connected health offerings of telemedicine, IoMT, and robotics industry.

All these forms of connected health technology will be critical to increase efficiencies, lower costs, and address health care workforce capacity issues. Furthermore, lack of intrinsic security measures can mean that health data on IoT devices may be vulnerable to threats, such as relying on a single gateway for data to be breached or failure to validate access or secure data when exchanged [71]. Blockchain has the potential to address these challenges by enabling the patient to monitor and control access and security to their data (including through the use of private encryption keys or smart contracts) that would ensure a higher level of autonomy, privacy, and control [71,72]. From a data integrity perspective, once recorded, the data in any given block cannot be altered retroactively without alteration of all subsequent blocks, rendering data tamper evident, and better securing health care data for purposes of analysis, insights into patient compliance and treatment, and also potentially enhancing reimbursement processes [73].

Blockchain’s potential potentially extends to Japan’s growing landscape of telehealth expansion. Research indicates that the focus areas for telemedicine development in Japan include increasing access to health care for rural and remote communities, enabling telemedicine in home care, and use for prevention and lifestyle modification [74]. Hence, the potential for blockchain-enabled telemedicine services, such as those being piloted in London, UK by the company Medicalchain, has the potential to secure telemedicine data (including Web-based and video consultations), create linkage with EHR data with access requests being recorded on a distributed ledger, and also enable payment via cryptocurrencies [75]. Japan’s potential for telehealth expansion coincides with recent deregulation of the telemedicine industry, where health insurance can now be used for telemedicine consultations [76]. The MHLW has also expressed its overall support of telemedicine in its proposal *Japan Vision: Health Care 2035* with Japanese health care startups actively providing remote consultation services for different conditions to Japanese health care institutions and their patients [76].

Additional opportunities for the mHealth and IoMT blockchain market are also emerging, as Japan represents one of the world’s largest markets for medical devices (valued at $33.3 billion in 2013) and as it represents a major export market, largely driven by its aging population that relies on devices for health maintenance and treating quality of life and age-related conditions [31]. Blockchains can enable interconnection of smart devices to collect health care data (including in the acute care setting, in the outpatient setting, and for home care) while also verifying the identity and provenance of data that may originate from multiple IoT-enabled sources [41]. Demonstrating early experimentation in this area of mHealth, Susmed Inc published a paper in 2017 in *JMIR mHealth and uHealth* describing the development of a blockchain-based tamper-resistant mHealth system using a smartphone app to deliver cognitive behavioral therapy for insomnia [41]. This in-country–driven innovation represents a unique combination of using blockchain as an mHealth intervention while also better securing data for use in clinical research [41].

Finally, Japan also has a robust robotics industry, including growing interest in the health care sector where robots are being tested for use in elderly care to address senior care workforce shortages, with projects enjoying support from the Japanese government [77-79]. In this context, blockchain has been proposed as a solution to better distribute and secure information for robotic swarm operations and make them more efficient, representing a potential frontier technology application for blockchain in health care [80,81].

### Decentralized Implementation?

Although Japan’s health care system has a centralized UHC structure, health blockchain implementation in Japan will likely be decentralized, meaning that it will not at first take an integrated approach. Although it may be ideal that all the blockchain use cases described above could be integrated into a single national blockchain technology framework and governance architecture, it is more likely that early implementation will occur through deployment of special-purpose blockchains specific to discrete health care challenges.

Our review finds that private and public sector blockchain use cases can have different goals and design elements (eg public vs private or consortium-based designs, differing permissions and privacy needs, varying forms of data integration and sharing, and may require different regulatory approaches). Hence, uniquely situated private sector versus public health sector needs may necessitate customized and adaptive policy making in combination with technical standard setting to better enable interoperability and shared benefit. Importantly, even in a centralized health system similar to Japan, one size will not fit all and Japan health blockchain deployments will need to be *fit-for-purpose* for specific health care challenges and the needs of their different local, provincial, and national-level stakeholders [3].

## Conclusions

Although Japanese health care blockchain use cases hold promise, progress in research and development, financial investment, and eventual deployment will require national government buy-in (as Japan’s health system is largely publicly funded) coupled with adaptive policy making to ensure blockchain technologies are incentivized and regulated correctly. Fortunately, the Japanese government is already making efforts to actively explore innovative and disruptive technologies through a regulatory sandbox system under the Cabinet Secretariat [82,83]. This system allows domestic and foreign organizations to apply for demonstration and evaluation of new technology, such as blockchain and IoT, without being subject to existing regulations while also opening up the possibility for future deregulation measures [82]. In fact, there are a few Japanese blockchain companies already taking advantage of the regulatory sandbox, including Susmed, Inc, which has 2 blockchain pilot projects for a clinical data management system [40].

Furthermore, leading Japan’s efforts in new technology spaces is the Cross-ministerial Strategic Innovation Promotion Program (SIP). The SIP is a national project for science, technology, and innovation supported by Japan’s Cabinet office. As an example of some of their efforts in emerging technology, the SIP is engaged in projects to establish artificial intelligence and big data solutions in hospitals to serve patients with automated diagnosis and treatment options through a commitment of $20 million in funding for 2018 [84]. One of the goals of the SIP is to establish a medical database with strong security, which is called subtheme A. Interestingly, according to the draft for subtheme A, blockchain technology will be explored as a potential solution for this project area. However, the Japanese government also hinted at some practical barriers including concerns that the costs of blockchain systems are still unknown and that there is a shortage of qualified blockchain developers. These real-world challenges have the potential to impede future government blockchain adoption in the health sector despite emerging policy support [85].

Collectively, government efforts and early commercial interest present an opportunity for shared decision making to develop technical standards around blockchain complemented by efforts of the SIP and regulatory sandbox regime. In fact, organizations such as the Institute of Electrical and Electronics Engineers (IEEE) Standards Association are working on a series of technical standards focused on distributed ledger technology use in the health care and life and social sciences (P2418.6), IoT (P2418.1), and applications in government (P.2418.8). Japan’s own IEEE Japan Council and its affiliated sections could contribute to development of these distributed ledger technology standards that could also be localized for Japan’s specific health, industry, and technology sector needs. This could also be coordinated through regulatory and policy coordination with the MHLW as the lead health agency and acting in conjunction with other nonhealth agencies working to encourage adoption of innovative technologies.

In addition, lessons for the future of Japan’s health blockchain can also be learned from the maturation of the financial services technology (also known as fintech) sector in the country. Blockchain’s earliest and most popularized use cases have arisen from cryptocurrencies, and more specifically bitcoin. Not surprisingly, Japan’s most mature sector for blockchain adoption has been fintech with several ongoing initiatives by Japanese banks and financial institutions to create cryptocurrencies and decentralized digital currency marketplaces and exchanges [86,87]. For example, Mitsubishi UFJ Financial Group, the largest bank in Japan, is planning on launching a bitcoin and cryptocurrency exchange targeting institutional and retail investors. In addition, Oversea-Chinese Banking Corporation, a bank in Southeast Asia, has carried out successful pilots of payment transactions using blockchain technology [88].

Overall, these fintech blockchains are designed to show that traditional functions of the financial system can be reliably executed by decentralized networks and in doing so speed up financial settlements while also raising the prospect of new financial system design [89]. However, there have been calls for regulation and taxation of fintech-related activities including blockchain, which include changes to Japan’s Financial Services Agency [90]. Hence, these early adoption challenges for fintech in Japan raise important questions regarding future adoption and regulation of blockchain technologies in other industries that are highly regulated and have privacy considerations such as health care.

Importantly, any Japan health blockchain strategy has to take into account the national and public health characteristics of Japan’s health system in addition to the unique challenges it faces with its aging society, concerns of health care overutilization, and shortage in its health care workforce. One future approach may be to design Japan’s health care blockchains as part of a Whole-of-Government Approach similar to Estonia where an electronic, national, government-backed blockchain is being used in the health care sector [91]. Regardless of the strategy, Japan’s current health care problems are acute, and its future health care challenges cannot be solved by a single solution, as technology is only one potential component of addressing complex population health challenges. In this sense, blockchain technology can act as an important enabler for technology and governance solutions for 21st-century Japanese health care, but only if it is localized, fit-for-purpose, and meets the needs of the Japanese people.

## Appendix

Multimedia Appendix 1 Summary of Japan health blockchain use cases.

## Abbreviations

eHealth: electronic health
EHR: electronic health record
GDP: gross domestic product
HIE: health information exchange
IEEE: Institute of Electrical and Electronics Engineers
IoT: Internet of Things
IoMT: Internet of Medical Things
JCQHC: Japan Council for Quality Health Care
mHealth: mobile health
MHLW: Ministry of Health, Labour and Welfare
OECD: Organisation for Economic Co-operation and Development
SIP: Cross-ministerial Strategic Innovation Promotion Program
IT: information technology
UHC: universal health coverage
