# Supplementary material for: Examining the Potential of Blockchain Technology to Meet the Needs of 21st-Century Japanese Health Care: Viewpoint on Use Cases and Policy
Source: J Med Internet Res. 2020 Jan 9;22(1):e13649. doi: 10.2196/13649 (PMC6996742; doi:10.2196/13649)
Supplement: Multimedia Appendix 1 [file jmir_v22i1e13649_app1.docx]

**Multimedia Appendix 1. Summary of Japan health blockchain use cases.**

| **Use case** | **Description** | **Specific challenge(s) addressed** | **Example** |
| --- | --- | --- | --- |
| Clinical research | Validate and manage clinical research data | Verifying clinical trial data; Electronic recruitment; clinical data monitoring; patient registries; and combating falsification of clinical data | Takeda cochairing Global Commission on rare disease diagnostics with announced blockchain projects; Susmed collecting data via mHealth^a^ application in regulatory sandbox; Japan Medical Association collecting Diabetes Patient registry |
| Supply chain | Establish integrity and trust in pharmaceutical supply chain | Enable regulatory adherence to track-and-trace requirements and combat falsified and substandard medicines | INDETAIL in Sapporo piloting dead stock clearing system for pharmacies in Hokkaido |
| EHRs^b^ | Improve integration of EHRs and reduce costs or waste | Enable sharing and portability of health care data; create verified provider and patient directories; linkage to My Number social security system; combat social security fraud; and enable data aggregation for research purposes | Arteryex collecting medical data from hospitals; Estonia operating electronic health system with single national EHR nationwide; Taipei Medical University launch of blockchain solution for patient referral and EHR integration |
| Telemedicine | —^c^ | Enhance access to health care services in rural and remote communities and creating linkage with EHRs and requests for data access | TRIART in Fukuoka Japan creating a system for rural telemedicine in Myanmar; Estonia operating teleradiology through a national image database; Medicalchain pilot in London |
| Internet of Things or Internet of Medical Things | Enable connected health through mHealth and connected devices | Enable devices and mHealth that can provide better home care services, health maintenance, and treating quality of life and age-related conditions; verify and better secure health data collected on multiple devices; and create tamper proof logs of maintenance of medical devices | Susmed Inc’s published research using blockchain for mHealth system for insomnia |
| Insurance | Reimbursement procedure | Patient authorization of medical data transfer from hospital to insurance company | Tokyo marine and Asou hospital in Kyushu |
| Local government | Citizen data | Create verifiable public records | Ichikawa city in Chiba and Kobe city in Hyogo |
| Robotics | Enable robotics to improve inpatient and home care | Robotics to help address elderly care and the need to address care workforce shortages | Research on blockchain application to robotic swarm operations |

^a^mHealth: mobile health.

^b^EHRs: electronic health records.
